# Controversial Regulation of Gene Expression and Protein Transduction of Aquaporins under Drought and Salinity Stress

**DOI:** 10.3390/plants9121662

**Published:** 2020-11-27

**Authors:** Lucía Yepes-Molina, Gloria Bárzana, Micaela Carvajal

**Affiliations:** Aquaporins Group, Centro de Edafologia y Biologia Aplicada del Segura, CEBAS-CSIC, Campus Universitario de Espinardo-25, 30100 Murcia, Spain; lyepes@cebas.csic.es (L.Y.-M.); gbarzana@cebas.csic.es (G.B.)

**Keywords:** aquaporins, abiotic stress, gene expression, post-translational modifications, turnover, trafficking, plasma membrane

## Abstract

Enhancement of the passage of water through membranes is one of the main mechanisms via which cells can maintain their homeostasis under stress conditions, and aquaporins are the main participants in this process. However, in the last few years, a number of studies have reported discrepancies between aquaporin messenger RNA (mRNA) expression and the number of aquaporin proteins synthesised in response to abiotic stress. These observations suggest the existence of post-transcriptional mechanisms which regulate plasma membrane intrinsic protein (PIP) trafficking to the plasma membrane. This indicates that the mRNA synthesis of some aquaporins could be modulated by the accumulation of the corresponding encoded protein, in relation to the turnover of the membranes. This aspect is discussed in terms of the results obtained: on the one hand, with isolated vesicles, in which the level of proteins present provides the membranes with important characteristics such as resistance and stability and, on the other, with isolated proteins reconstituted in artificial liposomes as an in vitro method to address the in vivo physiology of the entire plant.

## 1. Introduction

Aquaporins are regulated to deal with variations in the amount and availability of water, since they are channels that allow water transport across cell membranes [1]. They are regulated at multiple levels (for instance, during transcription and translation or through post-translational modifications) to modulate the trafficking, gating, or degradation/turnover [2]. This regulation is key to maintain cellular homeostasis under stress conditions, and, although our knowledge of plant aquaporins has experienced a tremendous increase over the past 20 years, different aspects of this regulation, especially in stressful conditions, are not yet known. With regard to aquaporins, the complexity of the response of plants to stress is due, on the one hand, to the multiple levels of regulation and, on the other, to the great variety of isoforms. It is important to highlight the discrepancies between messenger RNA (mRNA) and protein levels revealed in different studies over the years. A point of view that takes into account the translational modifications [3] will be key to understanding and explaining these types of discrepancies. It has been and will be crucial to address this research from the in vitro angle. In vitro methods led to the discovery of the first aquaporins [4] and revealed the structure of these proteins [5], among other important aspects. Furthermore, the importance of the lipid environment and the plasma membrane (PM) as a whole remains largely unexplored as far as aquaporins are concerned. Indeed, modification of the lipids, which appears under stress [6], can induce changes in intrinsic membrane proteins [7]. Therefore, the study of aquaporins should be approached from this perspective.

This review is mainly focused on salt and drought stress since they are two widespread environmental stresses. Soil salinity and drought affect plant growth and productivity and, therefore, are a problem for agriculture [8]. These two stresses, in many aspects, can be studied in parallel because both involve a reduction in water uptake and, thus, a net plant water loss; hence, aquaporins are important to the understanding of the response of plants to these stresses [9]. Among the distinct types of aquaporins, plasma membrane intrinsic proteins (PIPs) are located mostly in the plasma membrane (PM) [10] and have an important role in maintaining water transport in cells under normal and stressful conditions. Moreover, PIPs are the most studied aquaporins and certain discrepancies have been found, while many questions remain to be answered.

In this review, we highlight the main levels of PIP aquaporin regulation under drought and salt stresses, the discrepancies between these levels in different studies, and the key role of the PM as a whole in this regulation. The aim is to reinforce the importance of studying the different levels of regulation together and to complete our knowledge of the regulation of aquaporins under abiotic stress. Additionally, we point out important aspects of aquaporin regulation that have been revealed in different types of in vitro studies performed to address the in vivo physiology of the entire plant.

## 2. Aquaporins under Salinity and Drought

### 2.1. General Features

There are different types of abiotic stress (salinity, radiation, drought, floods, extreme temperatures, heavy metals, etc.) that usually cause loss of yield in major crop plants worldwide [8,11]. Therefore, detailed studies in this field are important, and numerous works have been carried out with the aim of facing up to abiotic stresses [12]. Despite the fact that these stresses have different origins and, therefore, distinct effects on the soil and on plant physiology, they all, as a primary effect, alter water uptake and, thus, have a high impact on plant–water relations [13]. Water transport from the soil into the roots and then through the entire plant, with subsequent evaporation to the atmosphere, is crucial for the development of essential physiological activities. This transport is a constant flux, based on three different pathways: (1) apoplastic, (2) symplastic, and (3) transcellular [14].

Therefore, the first response of plants to stress is to maintain their water homeostasis, which is essential to deal with environmental stress and to maintain plant development [15,16]. One of the mechanisms via which cells can maintain this homeostasis under stress conditions is the enhancement of water passage through membranes, in which aquaporins have the main role [9,10]. It has been reported that these proteins play key roles in osmoregulation [17], root hydraulic conductivity (Lpr) [18], leaf hydraulic conductivity [19], and transpiration [20].

Aquaporins are transmembrane proteins belonging to the major intrinsic protein (MIP) family. They have a molecular mass between 25 and 30 kDa, assemble into tetramers, and constitute channels in the cell membranes. These channels were first characterised by their transport of water across membranes, but it has been reported that aquaporins also transport small neutral solutes (urea, silicon, boron, hydrogen peroxide) or gases (ammonia and carbon dioxide) [1]. At the structural level, these proteins have six transmembrane α-helices and five connecting loops. In the two cytoplasmic loops appear two highly conserved Asn–Pro–Ala (NPA) motifs [21]. Furthermore, four conserved residues form a typical aromatic/arginine (Ar/R) constriction that functions as the main selectivity filter. Regarding their classification, aquaporins in plants are placed in seven subfamilies according to their intracellular locations and sequence similarities: (1) plasma membrane intrinsic proteins (PIPs), (2) tonoplast intrinsic proteins (TIPs), (3) nodulin 26-like intrinsic proteins (NIPs), (4) small, basic intrinsic proteins (SIPs), (5) GlpF-like intrinsic proteins (GIPs), (6) hybrid intrinsic proteins (HIPs), and (7) uncategorised X intrinsic protein (XIPs) [22].

Regarding the role of aquaporins in the maintenance or restoration of water homeostasis under abiotic stress, particularly drought and salinity, numerous studies have been carried out. These include research describing the behaviour of aquaporins under stress conditions from the point of view of gene expression and protein levels [23,24,25]. On the other hand, there are multiple investigations that include the overexpression of different aquaporins in order to elucidate their role in the response to salinity and drought stress [26,27]. Under these stress conditions, differences in the responses of aquaporins have been shown among different aquaporin homologues in diverse plants [28], among cultivars of the same plant species with different stress tolerance strategies [29], and between tissues [30]. Indeed, root transcriptomic analysis under drought stress showed both up- and downregulation of different PIP, TIP, and NIP homologues, while different homologues responded differently depending on the intensity and nature of the stress applied [10].

### 2.2. Discrepancies between the mRNA and Protein Levels of PIP Aquaporins

Aquaporins have been widely studied for years; however, there has been little investigation relating aquaporin expression and protein synthesis in the same study. There are several works that showed discrepancies between the expression of aquaporin genes and the number of aquaporin proteins present under abiotic stress conditions (Table 1), especially for PIPs. An understanding of the relationship between mRNA and protein levels is essential for integration of the physiological functions of aquaporins.

In the study carried out by Kammerloher et al. [31], in which the existence of water channels in the PM of plant cells was confirmed, these discrepancies first appeared. In this work, *Arabidopsis thaliana* plants were subjected to drought (0.6 M mannitol for 4 h) and changes in the PIP gene expression and protein levels were analysed. Different PIP genes (PIP1a, PIP1b, PIP1c, PIP2a and PIP2b, which correspond to *AtPIP1;1*, *AtPIP1;2*, *AtPIP1;3*, *AtPIP2;1*, and *AtPIP2;2*, respectively, according to Johanson et al. [38]) were analysed, and none of them were induced or repressed by the water stress. However, the amount of PIP in the PM increased twofold. At this point, there were two possible explanations for these discrepancies. On the one hand, the increase in *PIP* gene expression may have occurred during an earlier period of time and, 4 h after applying the stress, the mRNA level had returned to the initial levels and the amount of protein corresponding to that early overexpression response was detected. On the other hand, in *A. thaliana*, 13 PIP aquaporins have been described and only five were analysed individually at the mRNA level, while the protein content was determined using a nonspecific antibody. Therefore, it may have been other isoforms, apart from those analysed, that responded to the stress.

After this, other studies were carried out on *A. thaliana* to elucidate the regulation of aquaporins under drought stress. Jang et al. [39] analysed the expression of all PIP aquaporins genes in *A. thaliana* under drought (applied as 0.25 M mannitol) in the long term (2 days), as well as in the short term (4 h). A general decrease in the expression of *PIPs* in leaves and roots after 2 days of treatment was reported, except for *AtPIP1;3 AtPIP1;4*, *AtPIP2;1*, and *AtPIP2;5*, whose expression increased. On the other hand, after 4 h of drought treatment, almost all the *AtPIP1s*, as well as *AtPIP2;1* and *AtPIP2;5*, showed increased expression, while that of almost all the *AtPIP2s* and *AtPIP1;5* decreased [39]. This study contrasted with that of Kammerloher et al. [31], in which the expression of none of the PIP genes analysed changed. These differences could be due to the differing intensities of the stress; in Jang et al. [39], the intensity of the stress was lower (0.25 M mannitol versus 0.6 M mannitol). However, a differential expression appeared, compared to the control conditions, in both these experiments with *A. thaliana* plants. Moreover, another important factor may be the advancement in the technology used, since the second study was carried out in 2004 and the first in 1994. Nevertheless, an insight into the post-transcription events is not possible because the amount of PIP in the PM was not analysed in this study [39]. A later study of aquaporins in *A. thaliana* under a 12 day drought stress [37] revealed that the gene expression and protein levels showed a positive correlation in the aerial part. In this case, drought, applied by not watering, produced a general decrease in the mRNA level of all *PIP* genes, except for *AtPIP1;4* and *AtPIP2;5*, as in the study described previously [39]. Regarding the amount of protein, determined by Western blot with nonspecific antibodies (anti-PIP1s and anti-PIP2s), a decrease in PIPs was determined after 12 days of drought stress. When the watering was restored, the mRNA level recovered, but this increase was not reflected in the amount of protein. The authors attributed these discrepancies to a general time lag between transcription and protein synthesis and/or a negative translational regulation of aquaporin transcripts [37]. The use of nonspecific antibodies to detect different aquaporins is a limitation that has appeared in many papers over the years and that could affect the interpretation of the results.

Conflicting data regarding the transcriptional response of *PIP* genes and the abundance of PIPs have also been reported in studies carried out with *A. thaliana* under salt stress. It is noteworthy that, as with the drought studies, some discrepancies between studies were also found in work on aquaporins under salinity. Although it is difficult to compare studies due to differences in the length and intensity of the stress, some general considerations can be stated. In [39], a study with *A. thaliana* was conducted under salinity (150 mM NaCl for 24 h). In the aerial part, the expression of most PIP aquaporins did not change—except for *AtPIP2;2* and *AtPIP2;3*, whose expression increased, and *AtPIP2;6*, whose expression decreased. In the roots, something different occurred; the aquaporins whose expression changed were principally PIP1s, specifically *AtPIP1;1*, *AtPIP1;2*, and *AtPIP1;3* (for which it increased) and *AtPIP1;5* (for which it decreased). Among the root PIP2 aquaporins, only the expression of *AtPIP2;7* changed, showing an increase. Unfortunately, no protein measurements were performed in this study. There are no other studies in which the aerial part was analysed in these terms and there are no other works where the results obtained were similar to those described here. For example, in a study conducted in *A. thaliana* grown under conditions of salinity similar to those used in the study described above (100 mM NaCl for 24 h), a general decrease in *PIP* genes expression was shown [23]; only the expression of *AtPIP2;3*, *AtPIP2;6*, and *AtPIP2;8* did not change. It is difficult to find an explanation for this and, hence, it is necessary to focus on the differences between the two studies. In the first study described, 150 mM NaCl was applied to 20 day old plants, whereas 100 mM NaCl was applied to 14 day old plants in the second study. In the work carried out by Boursiac et al. [23], the amount of protein was also analysed 24 h after the treatment commenced, and the results showed a correlation between the mRNA levels and the amounts of PIP aquaporins in the PM, as both decreased. However, when the amounts of PIP aquaporins in the PM were analysed after long-term stress application (100 mM NaCl for 10 days), the amounts of PIP1 were similar to the control, while, for PIP2 aquaporins, the decrease continued [40]. In this study, the expression levels were not determined and, therefore, it is difficult to correctly interpret what is happening. Studies carried out in the short term (1 h and 4 h) with 10 and 100 mM NaCl showed a decrease in the expression of most *PIP* aquaporin genes, with the exception of *AtPIP2;6*, which in almost all cases showed a different behaviour [41,42]. There are not many published works in which a complete analysis of both the levels of expression of *PIP* genes and the abundance of PIP aquaporins was performed in relation to different intensities and durations of a stress. Hence, more specific experiments are needed to determine how the aquaporin levels in a model plant such as *A. thaliana* respond to salinity and, thereafter, to extend this knowledge to other species. Recently, Pou et al. [34] published a complete study of *AtPIP2;7* in *A. thaliana* with the objective of clarifying the conflicting data from other studies, such as those described above, of at least one of the PIP aquaporins. In this work, AtPIP2;7 was characterised at the gene and protein levels under salt stress (2 and 4 h at 150 mM NaCl). Salt stress triggered *AtPIP2;7* transcriptional repressions, but the amount of protein did not change after 4 h of treatment.

**Figure 1:**
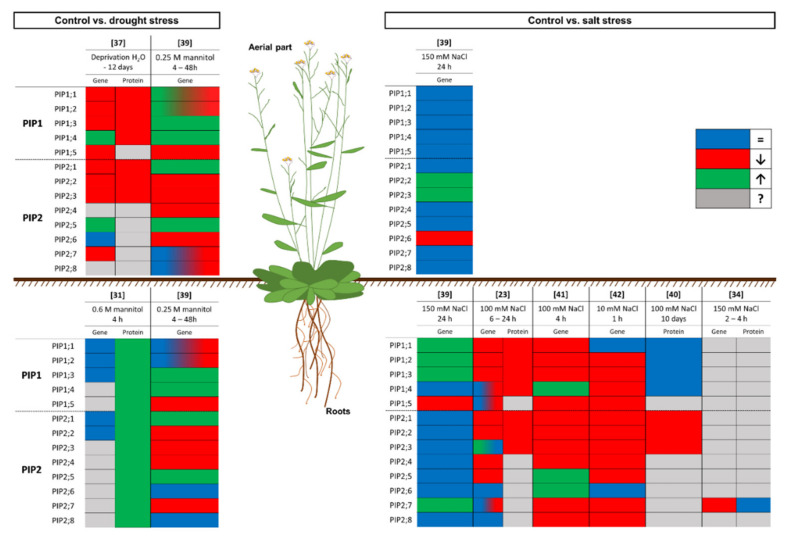
Graphical summary of the response of the model plant *Arabidopsis thaliana* to drought and salinity stress at the gene expression and protein levels. The box colour indicates the response of the plant compared to control conditions: the gene expression or protein amount does not change (blue), decreases (red), or increases (green); or unknown data (grey). Numbers in brackets refer to the related bibliography.

The results of the experiments carried out on *A. thaliana* show a downregulation of the expression of most aquaporins under drought or salinity stress, to limit water loss and induce stomatal closure [23,39,43]. Additionally, these experiments are a window into the complex system of regulation of aquaporins in plants subjected to water-related stress. Figure 1 shows a graphical summary of the response to water stress regarding the expression levels of *PIP* genes and the abundance of PIP in the PM in *A. thaliana*, which has been the major model plant in the past three decades [44]. Furthermore, it is important to point out that most of the work in this sense was conducted in the root, because it is the first organ to come into contact with the stress.

In species other than *A. thaliana*, there have been numerous studies where dissimilarity between the changes in mRNA and those in the protein levels for PIP aquaporins was reported. In *Zea mays* under drought stress, lasting 4 days (short-term drought) or 12 days (sustained drought), discrepancies between the mRNA and protein levels were reported [33]. In fact, *ZmPIP1;2* and *ZmPIP2;4* expression decreased after 4 days but the ZmPIP1;2 and ZmPIP2;4 protein levels were not reduced. However, a decline in the protein amount was detected after 12 days. Therefore, there was a correlation but only in the long term. On the other hand, this correlation was not found for ZmPIP2;1 and ZmPIP2;2 because the expression level was reduced after 4 days, but the protein level did not change after 4 or 12 days. This could be related to regulation at the functional activity level, through gating regulation to decrease water permeability, before the reduction of the level of expression was effective at the cellular level.

Suga et al. [32], in an experiment conducted with radish under drought (0.3 M mannitol for 6 h) and salinity (150 mM NaCl for 6 h), showed some discrepancies between the *PIP* mRNA and PIP protein levels. *RsPIP2;1* increased 1 h after the mannitol treatment and then decreased gradually; however, this increase was not reflected in the amount of RsPIP2;1 protein, which gradually decreased after the treatment. At first glance, this result could be explained by a low translation rate and/or a rapid turnover of PM or protein degradation. However, the same work showed a different behaviour of *RsPIP2;1* in radish under short-term (6 h) salt stress, since the expression of *RsPIP2;1* did not change, but the protein amount increased [32]. This pattern is similar to that reported by Kammerloher et al. [31] and described above for *A. thaliana* under drought stress; the mRNA levels did not change, but the protein increased. This could be due to accelerated trafficking, to redistribute aquaporins from reservoirs located in other organelles to the PM without the need to increase gene expression. For the same isoform (*HvPIP2;1*) in barley subjected to salt stress (150 mM NaCl) for 2 days, its expression decreased and was not correlated with the amount of protein, since that did not change [35]. The results reported in these studies show a regulation of aquaporins only at the protein level, since the increase in the short term was not due to mRNA translation and could have been due to post-translational modification or a redistribution of aquaporins in the cells. On the other hand, in the longer term, the mRNA level decreased, possibly due to inhibition by the redistributed aquaporins.

Additionally, other kinds of discrepancies have been reported. Muries et al. [24] showed that, in broccoli plants grown with 80 mM NaCl for 15 days, the expression levels of *PIP1* and *PIP2* decreased while the amounts of PIP1 and PIP2 protein in the PM increased. Furthermore, this increase in the protein amount was reported previously by López-Pérez et al. [6]. Although, in this study, some information could have been masked due to the use of nonspecific primers and antibodies, the explanation could be that the accumulation of the encoded protein inhibited the mRNA synthesis [45]. Furthermore, the importance of the stress intensity in the appearance or not of discrepancies between mRNA and protein levels was patent in work carried out in barley with HvPIP2;1 [35,36]. In this work, 150 mM and 200 mM NaCl applied for 2 days decreased the *HvPIP2;1* expression, but the protein amount only decreased with the higher concentration of NaCl.

## 3. PIP Trafficking and Turnover under Drought and Salt Stresses

The discrepancies reported between the mRNA and protein levels of PIP aquaporins suggest that the synthesis of aquaporins mRNA could be modulated by the accumulation of the corresponding encoded protein and the existence of post-transcriptional mechanisms that regulate PIP trafficking, gating, and turnover. Numerous studies, such as those listed above, have been conducted to understand the transcriptional regulation of PIPs in response to abiotic stress. Nevertheless, our knowledge about the post-translational regulation mechanisms that modulate the delivery of PIPs to the PM and their activity remains scant.

Although PIP aquaporins are described as being in the PM, these proteins play a major role in numerous essential processes in cells, including the response to salt and drought stress [9]. Therefore, it is logical that the trafficking, subcellular location, and turnover of these proteins are complex and highly regulated overall, especially under stress conditions [46], to deal with the resulting water stress.

In plants, there are two main pathways for protein trafficking: (1) the secretory pathway, which transports new PM proteins from the endoplasmic reticulum (ER) to the Golgi apparatus and finally to the PM or extracellular space, and (2) the endocytic pathway, through which PM-localised and extracellular factors are transported to endosomal compartments for their recycling [47]. In these pathways, the transport is carried out by vesicles, whose fusion with the target membranes is mediated by syntaxins, which are SNARE (soluble *N*-ethylmaleimide-sensitive factor attachment protein receptor) proteins [48,49,50]. Membrane trafficking is essential to maintain cellular functions in homeostatic conditions during the physiological response and adaptation to environmental stimuli such as abiotic stresses [51,52]. The compartmentalisation and abundance of different macromolecules are precisely regulated during stress responses, and they depend on membrane trafficking, since most macromolecules are synthesised far from the sites where they carry out their functions [48].

To avoid water loss in the face of salt and drought stresses, it is crucial to regulate the water transport by modulation of the gating of aquaporins [53] or by altering the number of active aquaporins in the PM, since downregulation of the aquaporins genes is not enough. Hence, it is necessary to modulate the translated aquaporins *en route* (I) and the aquaporins already in the PM (II).

### 3.1. Aquaporins En Route (Secretory Pathway)

The subcellular location of PIPs is important to control the cell water permeability [54], and their final destination is reached through the secretory pathway, where different molecular actors have been reported to be necessary for regulation of the post-Golgi trafficking of PIPs to their final location (mainly the PM), through PIP1 and PIP2 hetero-oligomerization [55,56], ubiquitinization [57], or phosphorylation [58], or the presence of an ER diacidic export motif [59,60]. However, it is not completely clear how many of these factors are needed at one time or the inter-relationships between them for the PIP aquaporins to reach the PM. For example, Zelazny et al. [59] showed that the diacidic motif present in some isoforms, such as ZmPIP2;5, was not sufficient to export ZmPIP1;2 from the ER; therefore, other factors are necessary for it to reach its final destination. These factors are necessary to explain the response of plants to salt and drought stresses, since regulation at the gene level alone is not enough to explain some behaviours, such as an increase or a lack of change in the expression of aquaporins genes coinciding with a decrease in protein abundance after an abiotic stress [32]. For example, Prak et al. [61] showed the existence of a mechanism to reduce water transport quickly, without regulation of gene expression, which acts on PIPs *en route* to the PM. This is accomplished by blocking the trafficking of new PIPs from the ER to the PM, through dephosphorylation at key residues of the aquaporins and the accumulation of these proteins in intracellular structures to prevent them from reaching the final destination (the PM).

In addition to the factors listed above, SNARE proteins, which can interact with the membrane proteins cargos (such as PIPs) transported by vesicles and modulate their activity over timescales of minutes to hours [62], have been reported as a requirement for proteins to reach the PM [46]. In this sense, it has been shown in maize that the physical interaction between ZmPIP2;5 and ZmSYP121 was the key for the post-Golgi trafficking and the water channel activity of this aquaporin [63]. SYP121 is one of the most studied PM-resident syntaxins and is known to be involved in vesicle traffic between the Golgi apparatus and PM [64]. Moreover, under drought stress, the expression of both *AtPIP2;5* [39] and *NtSYP121* [65] was increased in the aerial parts of *A. thaliana* and *Nicotiana tabacum*, respectively. The higher expression of *NtSYP121* could be related to the need to enhance the trafficking of PIPs, such as PIP2;5, to the PM in the aerial part, since the upregulation of specific PIP isoforms was related to their involvement in gas and water exchange and stomatal behaviour [66,67]. Similar results were reported for PIP2;7 in *A. thaliana*; in this case, the interaction with SYP61 and SYP121 formed a SNARE complex mediating the post-Golgi trafficking of this aquaporin, the delivery to the PM, and the water permeability of the cell membrane [68].

The results obtained in these studies allow us to define a model including the SNARE activity necessary to regulate the water permeability of the membrane. This shows the complexity of the mechanisms required for PIPs to reach the membrane, with differences in the specific signal and the trafficking mechanism depending on the aquaporin isoform. This complexity could affect the delay seen between an increase in mRNA expression and that of the protein amount, or other discrepancies described above.

### 3.2. Aquaporins Already in the Plasma Membrane (Endocytic Pathway)

The discrepancies between changes in mRNA levels and protein amounts listed above could also be explained by a regulation based on turnover of the membranes, such as when there is a decrease in the mRNA level but the protein level does not change or when the mRNA level increases but the protein level decreases.

Under this assumption, in the first case, the turnover would be slow (a minimum of more than 4 h, according to the results shown by Pou et al. [34]) and, in the second, it would be fast (less than 1 h, according to the results shown, for example, by Suga et al. [32]).

Membrane turnover is controlled by endocytosis, involving invaginations of the PM, and endosomal trafficking to carry the membrane proteins cargo via endocytic vesicles back to the ER though the *trans*-Golgi network (TGN), for recycling, or to the vacuole, for degradation. These processes are essential because, together with exocytosis, they regulate the PM composition, which is key for cell survival and for appropriate development and physiological responses, including those against abiotic stresses [49,69]. Two main endocytic pathways have been described in plants, as well as in animals: a clathrin-dependent pathway and a raft-associated pathway (clathrin-independent) [70].

A combination of transcriptional regulation and post-translational modification of PIP aquaporins was reported as essential for the inhibition of *Lpr* in *A. thaliana* roots during 24 h salt stress (100 mM NaCl) exposure [23]. Short-term stress (minutes to an hour) decreased the expression of aquaporins genes, as an immediate reaction, but the protein amount diminished sometime later. Therefore, in the early phase of salt stress, a stimulus-dependent subcellular relocation of aquaporins was induced, and, after 2 h of stress application, the intracellular structures containing AtPIP1;2 and AtPIP2;1 (two of the most abundant PIPs in roots) appeared in root cells, as a rapid mechanism to decrease the amount of PIPs in the PM and consequently *Lpr* [23], a change that is essential to deal with salt stress. Subcellular trafficking of aquaporins as a response to an osmotic stress was first described in the ice plant (*Mesembryanthemum crystallinum*), when McTIP1;2 was redistributed to intracellular vesicles from the tonoplast [71]. In a further study, Boursiac et al. [72] described a relationship between signalling pathways triggered by reactive oxygen species (ROS) accumulated under abiotic stresses and internalisation and relocalisation of PIPs into intracellular structures, with the purpose of downregulating root water transport. In another study carried out with AtPIP1;2 and AtPIP2;1, using the fluorescence recovery after photobleaching (FRAP) approach, the exchange between the PM and intracellular pools was examined, and the PM aquaporin cycling in roots of *A. thaliana* was greater under salt stress than under resting conditions [73].

Another study to elucidate the PIP2;1 turnover in the face of abiotic stresses was carried out by Lee et al. [57] for drought-stressed *A. thaliana* plants. The ER-localised E3 ubiquitin ligase RM1H1 interacted with AtPIP2;1 at the ER and appeared to play a key role in the proteasomal degradation of PIP2;1 as a response to water stress.

Regarding PIP2;7, some studies reported a hypothetical model of regulation of the endosomal trafficking, endocytosis and degradation of this aquaporin by tryptophan-rich sensory protein/translocator (TSPO), which decreases the amount of this aquaporin in the PM under abiotic stress conditions [74]. TSPO is a multi-stress regulator whose expression is induced by the phytohormone abscisic acid (ABA). The study hypothesis states that, as the levels of ABA increase during water stress [75], the protein–protein interaction between TSPO and PIP2;7 in the ER and Golgi is involved in the vacuolar degradation of PIP2;7 through an autophagic pathway mediated by ABA. This would correspond to a response to short-term stress, as established by Bárzana et al. [76] in a recent review. This occurs due to the need to reduce the level of proteins already synthesised, since a decrease in gene expression is not sufficient to limit intracellular water transport and avoid dehydration. Therefore, most of the literature reports a decrease in root PIP abundance under these stresses [10]. Concerning PIP2;7, Pou et al. [34] showed that gene expression, but not the protein amount, decreased under short-term salt stress. These two studies conducted with AtPIP2;7 at the gene and protein levels together reveal the complex regulatory mechanism that is triggered under abiotic stress. It is possible that the need to interact with another protein (TSPO), which is overexpressed in these conditions, delays the decrease in PIP2;7 and, therefore, it is not detected by short-term measurements. In this regard, Ueda et al. [77] showed that other factors have a role in the internalisation of PIP2;1 from the PM to the vacuole under salt stress. In this study, two kinases (phosphatidylinositol 3-kinase (PI3K) and phosphatidylinositol 4-kinase (PI4K)) were described as being key in this process in saline conditions, together with clathrin-mediated endocytosis (CME). This contrasts with the previous work by Luu et al. [73], who described clathrin-independent endocytosis (CIE) as being involved in the recycling of PIP2;1 from the PM under salt stress, while CME was associated with non-saline conditions.

Recently, some of these assumptions, studied mainly in *A. thaliana*, were confirmed in tomato plants [78]; in this study, an increase in *Lpr* was found in salt-stressed plants when inhibitors of endocytosis and phosphatase were applied. These results suggest that the activity of PIPs under stress conditions depends on dephosphorylation and internalisation to a large degree. The reduction of *Lpr* should be due to the decrease in PIP activity and, as an immediate reaction to stress (1 h), water homeostasis is maintained through the modulation of aquaporins activity and, to a lesser extent, through the regulation of gene expression.

## 4. In Vitro Studies to Determine the In Vivo Physiology of Plant Aquaporins

Numerous studies have been carried out to find out how aquaporins are regulated under abiotic stress, especially stresses involving water stress, such as salinity or drought. These studies, some of which are described above, reveal that there are still many details to be discovered and that contradictory data exist. They also show that it is difficult to elucidate the behaviour of aquaporins in vivo due to the large number of aquaporin isoforms and the difficulty of studying one in particular without affecting the role of another. Moreover, aquaporins function mainly as channels for water; since this is the medium of all biological processes, aquaporins may participate in most of them.

In this sense, in vitro studies using PM vesicles isolated from different organs and plants, isolated aquaporins reconstituted in artificial liposomes, or aquaporin expression in oocytes are useful methods to elucidate details about the transcription and post-translational regulation of aquaporins. They also shed light on the gating (in fact, the first knowledge of plant aquaporins gating was obtained through in vitro studies using isolated plant membranes [79] or proteoliposomes [80]), turnover, and trafficking of these proteins under abiotic stresses. Important findings have been made using these methods. For example, the first aquaporin (CHIP28) was discovered when the expression of the *CHIP28* gene in oocytes from *Xenopus laevis* increased the osmotic water permeability, and this was reversibly inhibited by mercuric chloride (a water channel inhibitor) [4]. Furthermore, the measurement of the water permeability in PM vesicles from wheat indicated the presence of aquaporins [81].

The coincidence of a decrease in the expression of PIP aquaporin genes with an increase in the amount of PIP in broccoli plants subjected to long-term salinity stress [24] is a discrepancy already mentioned above and could be due to inhibition of mRNA synthesis by PIP. Further analysis of these results could not have been carried out without in vitro studies [6]. The experiments conducted with PM vesicles from roots of broccoli plants grown under salinity helped to reveal the state of the aquaporins in the PM and to elucidate why this discrepancy appeared. Therefore, when the osmotic water permeability (*Pf*) was measured, similar values were obtained for PM vesicles purified from NaCl-treated and control plants, indicating that aquaporin activity was not affected by salt stress. With all these data, it could be suggested that aquaporins accumulate in the PM to inhibit the expression of their corresponding genes, but they are inactive; therefore, there is no increase in water transport, which can be detrimental under salt stress.

Experiments conducted with PM vesicles are also useful to understand the role of the post-translational modification of aquaporins. In a study carried out with PM vesicles from broccoli roots, an increase in acetylation in PIP1 aquaporins under salt stress was reported, and this may be related to an enhancement of their stability, supported by the fact that *Pf* was more stable during storage for vesicles from NaCl-exposed roots [82]. In this case, the post-translational modification was not related with the functionality of the aquaporins, since acetylation was related principally to increasing the half-life of the proteins conferring resistance to derivative processes [83]. This makes sense in relation to the above, since salt-stressed plants try to reduce water uptake in general terms; however, in broccoli, an increase in aquaporins in the PM has been described under long-term salinity. Acetylation increases aquaporins stability and may be necessary, along with lipid modifications, to maintain an appropriate PM composition to cope with stress.

Phosphorylation is an important post-translational modification for aquaporins and is listed above as one of the modifications implied in the post-Golgi trafficking of PIPs to their final location, as well as in the gating of aquaporins. This modification has been studied for years using in vitro methods. Johansson et al. (1998) [84] demonstrated the activation of spinach leaf aquaporin SoPIP2;1 (formerly called PM28A), when expressed in oocytes, by the reversible phosphorylation of two residues of serine (Ser115 in cytosolic loop B and Ser274 in the carboxy-terminal region). In this study, the restriction of the water flow through aquaporins by dephosphorylation of these residues when the leaves suffered water deficiency (drought stress) was also suggested. Furthermore, to understand the gating mechanism based on phosphorylation, the structure of this spinach aquaporin was described and its water channel activity was assayed by reconstitution into proteoliposomes using *Escherichia coli* lipids [5]. In fact, in this study, the water channel activity of an aquaporin overexpressed in and purified from *Pichia pastoris* was reconstituted for the first time, which was a breakthrough in the functional and structural studies of these proteins. This method is very useful for the study, in both wildtype and mutant aquaporins, of specific residues involved in particular processes. SoPIP2;1 and mutants of SoPIP2;1 (in both Ser115 and Ser274) were overproduced to characterise the molecular details of channel gating due to the phosphorylation state, by resolving the X-ray structures in the open and closed conformations of the wildtype and mutants [53,85]. Törnroth-Horsefield et al. [53] also showed that the protonation of two residues (His193 in loop D and Asp28 in the N-terminus) maintained SoPIP2;1 in a closed state via an ionic interaction. These molecular structures provide a basis for future investigations into the molecular factors involved in PIP gating.

The latter finding is supported by the results provided by an assay carried out with PM vesicles from *A. thaliana* suspension cells, where the contribution of Ca^2+^ and H^+^ to the reversible inactivation of water channels was revealed [79]. According to this and using the method of aquaporins reconstitution in proteoliposomes, structure–function analyses were performed in other species to provide new insights into PIP gating by divalent cations and protons [80]. Water transport assays with wildtype and site-directed mutants of AtPIP2;1 (overproduced in *P. pastoris* and reconstituted into proteoliposomes) confirmed the blockage by H^+^ while also showing the inhibition of aquaporin gating by Ca^2+^, Cd^2+^, and Mn^2+^, representing a new finding since the inhibition occurred for an individual PIP isoform. This is unlike the report of Alleva et al. [86], where the heterogeneity of aquaporins in *Beta vulgaris* PM vesicles meant that it was not clear which isoform was responsible for the Ca^2+^-dependent inhibition of *Pf*.

These in vitro studies are interesting also to elucidate the functions in which the different isoforms of aquaporins are involved; studies of their expression, translation or trafficking in a context of abiotic stress could help to explain the discrepancies.

Liu et al. [87] showed that OsPIP1;1 has water channel activity in liposomes, and these data, together with those obtained from assays in planta, demonstrated the role of OsPIP1;1 in rice physiology, such as in seed germination or salinity resistance. The isoform *AtPIP1;1* is one of those whose expression increases in roots under salt stress [39], when in general a decrease in *PIP* expression has been reported. An increase in the expression of some *PIP* isoforms may mean that they are involved in mechanisms that promote stress resistance, as is the case with OsPIP1;1 in rice [87].

In another study conducted with SoPIP2;1, Kirscht et al. [88] showed the importance to the water transport function of the cysteine (C69) that is conserved among different PIPs in the first extracellular loop (loop A) of each monomer. Wildtype (WT) SoPIP2;1 and two C69 mutants were reconstituted in liposomes, and the mutants showed greater water transport. The authors suggested that this could have arisen because these mutations prevented the formation of the disulphide bridge between the monomers and increased the size of the central pore. Additionally, this study indicated that mercury, considered as a general aquaporin blocker, increased the water permeability of WT SoPIP2;1 and the mutants reconstituted in liposomes. Therefore, these conclusions open a new perspective in which mercury activates some aquaporins under specific conditions. Even though it does not have a relevant role physiologically, mercury is an important tool in the study of aquaporins [1] and this new knowledge could provide alternative opportunities to modulate and study water homeostasis in plants.

Recently, Wang et al. [89,90], by means of the reconstitution of purified aquaporins in artificial liposomes, established a method to determine the capacity of aquaporins isoforms to transport H_2_O_2_ in vitro, as well as reported the first PIP aquaporin (AtPIP2;4) structure from *A. thaliana*. The results provided by this research suggest a role of the loop regions in the specificity of the aquaporins. This, together with phosphorylation and protonation [53], provides a very detailed model for the gating mechanism of PIP aquaporins regarding water and H_2_O_2_ transport.

The above examples are a demonstration that studies involving the functional incorporation of aquaporins into liposomes provide useful information about their functions and regulation. On the basis of all the above mentioned knowledge, we now consider the role that the PM as a whole must have in the regulation of aquaporins, including trafficking and turnover, as well as in their function.

In a study carried out in mammals, two isoforms of aquaporin-4 (AQP4), the most abundant water channel in the mammalian brain, were reconstituted into liposomes with different lipid composition to elucidate the relationship between the aquaporin and its lipid environment, the objective being to translate this information into in vivo conditions [91]. It was shown that, if the bilayer of the artificial liposomes is more compact and thicker, the water permeability of both isoforms is lower. This could have physiological effects, since aquaporins recruited in raft microdomains (thicker and less compressible than the surrounding membrane regions) [92] have decreased water permeability. This study indicates that raft microdomains are involved not only in aquaporin trafficking and turnover, but also in their functionality in water transport, which could be of key importance under water stress.

As with many others, it could be interesting to extrapolate this aspect to plants, although it requires further investigation. In this sense, in the study mentioned above, Kirscht et al. [88] suggested a role for lipids in the avoidance of water and proton leakage, since aquaporin structures have a lipid molecule in the central pore [93,94], which could be translated to the native environment. Moreover, previous in vitro assays showed how, depending on the membrane composition, the behaviour of aquaporins can differ. For example, barley HvPIP1;2 without co-expression with PIP2s did not increase water permeability in *Xenopus* oocytes, but this co-expression was not needed in yeast, which reveals that different membrane systems affect aquaporin functioning [95,96]. In the same way, NtPIP2;1 facilitated CO_2_ diffusion in artificial membranes but not in yeast [97,98]. The findings mentioned here concerning the lipid environment and aquaporins regulation suggest that alteration of PM lipids is important in the regulation of the permeability of aquaporins [99].

## 5. Concluding Remarks

**Figure 2:**
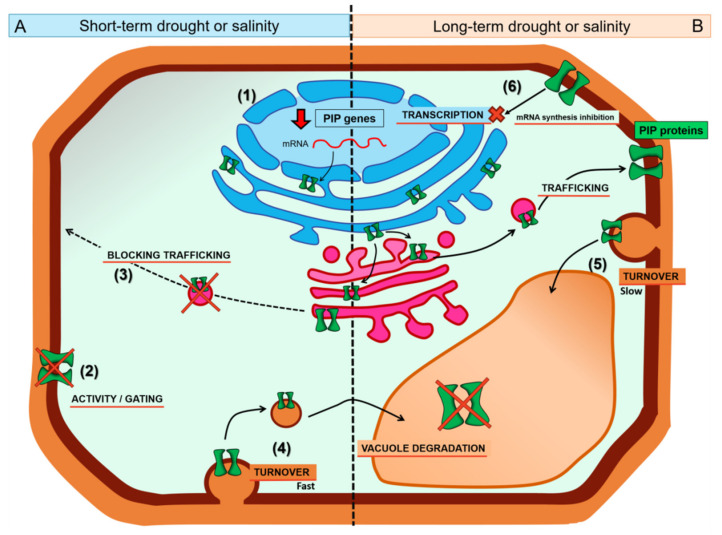
Graphical summary of the hypothetical regulation at cellular level of plant plasma membrane intrinsic proteins (PIPs) under short- (**A**) and long-term (**B**) salinity or drought stress. Most PIP genes decrease their expression under both conditions (1). Under short-term stress (drought/salinity), the functionality of the PIPs already in plasma membrane (PM) is repressed through gating or modifying their activity by post-translational modification (2), and the trafficking of PIPs in route to PM is blocked (3). Turnover of PIPs in PM would be fast under short-term stress (4) and slow with long-term stress (5). Under long-term stress (drought/salinity), PIPs could inhibit transcription of PIP genes (6) to promote a decrease in the amount of PIP.

In the response of plants to abiotic stresses such as salinity or drought, aquaporins have a key role. The regulation of these proteins has been studied in depth at different levels for years: transcription, translation, post-translational modification, trafficking, turnover, and gating. In this sense, many important aspects have already been described but many others have not yet been elucidated. The control of water flow through aquaporins consists of a fine and complex regulation. However, to determine the specific mechanisms involved at each level of regulation under salinity and drought stress, several parameters should be taken into account. It was revealed in this review that small differences in the experimental conditions can lead to contradictory results, making it difficult to compare different research findings. The differences in salt and drought tolerance between different species or varieties of plants should be borne in mind. Since the model plant *A. thaliana* is not particularly tolerant of these two stresses, the extrapolation of its results to other, commercial species should be done with care. Furthermore, the behaviour of aquaporins at the gene and protein levels depends on the intensity and/or duration of the applied stress, and the duration of the stress (short or long-term) is the crucial aspect influencing the level of regulation (Figure 2).

In this sense, we also showed that the modification of gene expression and the accumulation of protein in response to salinity or drought are not always correlated since the change in gene expression seems to be a short-term response, whereas protein regulation seems to be a long-term response. Therefore, an approach from a broader point of view will be important to explain these discrepancies, taking into account the role of other factors in the regulation of the amount and activity of aquaporins, to maintain or restore water homeostasis during abiotic stress. An interesting hypothesis, which needs more investigation in plants, is that the protein already translated initiates a signalling cascade that inhibits the transcription of the gene corresponding to the protein. This could explain the absence of a correlation between the amounts of mRNA and protein for certain isoforms of aquaporins under drought and salt stresses. Additionally, the lack of specific antibodies for the different isoforms of aquaporins is a limiting factor in the interpretation of the results, since some changes may be masked by other isoforms. Few studies exist where antibodies for specific isoforms were used. Thus, it is necessary to make an effort to advance the design of specific antibodies for the different isoforms of aquaporins in different plant species.

The use of in vitro methods (overexpression in oocytes, reconstitution in artificial liposomes, or the use of isolated membrane vesicles) has been and will be fundamental in the deepening of our knowledge about aquaporins. Information essential to the continued advance of our knowledge of aquaporins and their regulation, such as the three-dimensional (3D) structure or gating, should be obtained through in vitro methods.

Furthermore, the overall information collected together in this review shows that the regulation of both the quantity and the activity of aquaporins in the membrane should be the key role of gene expression. In order to continue adding to our knowledge of the specificities of aquaporins regulation under abiotic stresses, it is also essential to consider the PM as a whole, taking into account that modifications of the lipid environment will affect aquaporins.

## Figures and Tables

**Table 1:** Summary of the plant responses to salinity and drought stress at the gene expression and protein accumulation levels of plasma membrane intrinsic protein (PIP) aquaporins.

Gene Expression	Protein Amount	References
=	=	
	↑	[31,32]
	↓	[32]
↑	=	
	↑	
	↓	[32]
↓	=	[33,34,35]
	↑	[24]
	↓	[23,33,36,37]

No change, =; Increase, ↑; Decrease, ↓.
